# Novel physico-chemical diagnostic tools for high throughput identification of bovine mastitis associated gram-positive, catalase-negative cocci

**DOI:** 10.1186/1746-6148-10-156

**Published:** 2014-07-11

**Authors:** Lydia Schabauer, Mareike Wenning, Ingrid Huber, Monika Ehling-Schulz

**Affiliations:** 1 Functional Microbiology, IBMH, Department of Pathobiology, University of Veterinary Medicine, Veterinaerplatz 1, 1210 Vienna, Austria; 2 ZIEL, Research Center for Nutrition and Food Sciences, Microbiology Unit, Technical University Munich, Weihenstephaner Berg 3, 85350 Freising, Germany; 3 Bavarian Health and Food Safety Authority (LGL), Veterinärstraße 2, 85764 Oberschleißheim, Germany

**Keywords:** Bovine mastitis, FTIR spectroscopy, MALDI-TOF MS, Artificial neural network

## Abstract

**Background:**

The routine diagnosis of *Streptococcus* spp*.* and other mastitis associated gram-positive, catalase-negative cocci is still based upon biochemical tests and serological methods, which frequently provide ambiguous identification results. We therefore aimed to establish an accurate identification system for differential diagnosis of mastitis associated *Streptococcus* spp. and related species using biophysical techniques such as Fourier-transform infrared (FTIR) spectroscopy and MALDI – TOF/MS.

**Results:**

Based on a panel of 210 isolates from cases of bovine mastitis, an unsupervised FTIR spectral reference library was established and an artificial neural network (ANN) - assisted identification system was developed. All bacterial isolates were previously identified by species-specific PCR and/or 16S rRNA gene sequence analysis. An overall identification rate of 100% at species level for 173 strains unknown to the ANN and the library was achieved by combining ANN and the spectral database, thus demonstrating the suitability of our FTIR identification system for routine diagnosis. In addition, we investigated the potential of matrix-assisted laser desorption/ionization time-of-flight mass spectrometry (MALDI-TOF MS) for the identification of mastitis associated *Streptococcus* spp. and related bacteria. Using the Microflex LT System, MALDI Biotyper software™ (V3.3) we achieved an accuracy rate of 95.2%. A blind study, including 21 clinical samples from dairy cows, revealed a 100% correct species identification rate for FTIR and 90.5% for MALDI-TOF MS, indicating that these techniques are valuable tools for diagnosis.

**Conclusions:**

This study clearly demonstrates that FTIR spectroscopy as well as MALDI-TOF MS can significantly improve and facilitate the identification and differentiation of mastitis associated *Streptococcus* spp*.* and related species. Although the FTIR identification system turned out being slightly superior to MALDI-TOF MS in terms of identification on species level, both methods offer interesting alternatives to conventional methods currently used in mastitis diagnosis as both of them provide high accuracy at low operating costs once the instrument is acquired.

## Background

Bovine mastitis is one of the most frequent diseases in dairy cattle and causes major economic losses due to reduced milk quantity and increased costs of treatment [1]. Besides staphylococci, streptococci and other related gram-positive, catalase-negative cocci represent the most important causative agents [2]. Especially *Streptococcus uberis*, in particular, is frequently isolated from cows with subclinical intramammary infections. Generally, *S. uberis* is regarded as an “environmental” pathogen but cow-to-cow transmission has also been reported [3]. *Lactococcus garvieae, Lactococcus lactis* and *Aerococcus viridans* are occasionally isolated from raw milk, but their role in mastitis is still under debate. One reason for the lack of resilient and conclusive data on the significance and role of certain *Streptococcus* spp. and other gram-positive, catalase-negative cocci in bovine mastitis might be the methods currently used in routine diagnosis, which are prone to error and often provide equivocal results at species level (see e.g. [2]).

In veterinary diagnostic laboratories, the identification of gram-positive, catalase-negative cocci is still mainly based on biochemical tests and serological grouping. However, these methods are rather time-consuming, labor-intensive and may give uncertain results due to a lack of mastitis-associated species in database or misinterpretation [4]. Furthermore, Lancefield-group antisera do not react with every streptococcal species [5]. Identification at species level can be achieved by molecular biological methods but the spectrum of microorganisms that can be detected and identified simultaneously is limited [6]. In the light of the growing threat of antibiotic resistant bacteria, fast and proper identification systems are not only crucial for determination of the role of certain bacterial species in bovine mastitis but also for choosing the right therapeutic treatment [7]. For targeted therapies, fast, easy-handling and accurate identification methods, allowing the discrimination of bacteria at least at species level, are urgently needed.

Fourier transform infrared spectroscopy (FTIR) is known to be a non-destructive method that is able to identify microorganisms by whole-organism fingerprinting and differentiates at different taxonomic levels [8,9]. Absorption of IR light by all cellular components of intact cells, such as proteins, polysaccharides and lipids, results in characteristic infrared spectra that can be used for identification of unknown samples by comparing their spectra with those from spectral reference databases of known species (for introduction to FTIR see [10]). Due to its discriminatory power, FTIR is also suitable to discriminate closely related bacterial species and for typing of bacteria, especially when it is combined with chemometrics [9]. For instance, artificial neural network (ANN), a supervised pattern recognition technique, has already been employed for typing bacteria at subspecies level [11-13]. ANNs belong to systems of artificial intelligence and can be trained to solve identification problems by acquiring and storing knowledge of reference data for identifying unknown samples [14]. Furthermore, FTIR spectroscopy has been shown to be a suitable tool for epidemiological investigations and tracing contamination sources [15-17].

Another biophysical technique using spectral data for bacterial identification that is gaining increasing attention, not only in human but also in veterinary clinical diagnosis, is matrix-assisted laser desorption/ionization time-of-flight mass spectrometry (MALDI-TOF MS) [18-20]. It uses protein “fingerprints”, particularly of ribosomal proteins, for bacterial species identification [21,22].

In the present study, the potential of FTIR spectroscopy and MALDI-TOF MS for identification and differentiation of mastitis relevant streptococci and other gram-positive, catalase-negative cocci was assessed and the applicability of both methods for routine diagnosis was examined in a blind study.

## Methods

### Bacterial isolates

A total of 383 bacterial strains were included in this study. The strain collection comprised strains isolated from bovine subclinical and clinical mastitis, as well as strains from blood, food, and pharmaceutical indoor environmental monitoring. Reference strains for each species tested were obtained from the ‘Deutsche Sammlung von Mikroorganismen und Zellkulturen’ (DSMZ, Braunschweig, Germany) and the American Type Culture Collection (ATCC, Virginia, USA) (see Additional file 1). Isolates from bovine mastitis were provided by the Clinic of Ruminants, University of Veterinary Medicine (Austria), Animal Health Service (TgD) of Lower Austria, Upper Austria, Styria, Carinthia, quality laboratory Lower Austria, milk laboratory (MBFG) Wunstorf (Germany) and the Institute of Microbiology in Bellinzona (Suisse). Streptococci from food and pharmaceutical industry were used as “outgroup” strains. The latter isolates were obtained from the culture collection of Microbiology Unit, ZIEL, Technical University Munich (Germany). 210 isolates were used to train and develop the ANN; the remaining 173 isolates were used for validating the resulting ANN (Table 1).

**Table 1:** Species and number of strains used for the development of the ANN-assisted FTIR identification system

**Species**	**Training**	**Validation**	**Total**
Masitis associated *Streptococcus* spp. and related species			
(*n* = 12)			
*A. viridans*	13	4	17
*E. faecalis*	22		30	52
*E. faecium*	11		5	16
*L. garvieae*	15		2	17
*L. lactis*	16		7	23
*S. agalactiae*	18		10	28
*S. bovis*	14		5	19
*S. canis*	9		2	11
*S. dysgalactiae*	21		36	57
*S. parauberis*	10		1	11
*S. pyogenes*	16		7	23
*S. uberis*	26		64	90
Outgroup				
(*n* = 9)				
*S. equi*	5			5
*S. gallinarum*	1			1
*S. gallolyticus* subsp. *macedonicus*	1			1
*S. mitis*	1			1
*S. oralis*	1			1
*S. pneumoniae*	3			3
*S. porcinus*	1			1
*S. sanguinis*	3			3
*S. suis*	3			3
Total	210		173	383

Strains were divided into two subsets, a training set and a validation set (for details see Methods).

Samples derived from routine diagnostic laboratories were subjected to species-specific PCR, using the primers and methods listed in Table 2, in order to determine their unequivocal identification. For species where no specific primers were available as well as for all strains of the blind study 16S rRNA gene sequence analysis was carried out as described previously [23]. Sequencing was performed at LGC Genomics (Berlin, Germany) and sequence analysis was carried out using the EzTaxon-e server (http://eztaxon-e.ezbiocloud.net/; [24]).

**Table 2:** Oligonucleotide primers used for species-specific PCR

**Species**	**Oligonucleotide sequences (5′-3′)**	**Target gene**	**PCR-product (bp)**	**Reference**
*E. faecalis*	*ATCAAGTACAGTTAGTCT*	genes encoding D-Ala:D-Ala ligases	941	[25]
	*ACGATTCAAAGCTAACTG*			
*E. faecium*	*GCAAGGCTTCTTAGAGA*		550	
	*CATCGTGTAAGCTAACTTC*			
	*GTTGAGCCACTGCCTTTTAC*	16 S rRNA	252	[26]
*L. garvieae*	*AATGGGGGCAACCCTGA*			
*L. lactis*	*GTTGTATTAGCTAGTTGGTGAGGTAAA*		387	
*S. agalactiae*	*AACAGCCTCGTATTTAAAATGATAGATTAAC*	23S rRNA	866	[6]
	*TCCTACCATGACACTAATGTGTC*			
*S. bovis*	*CCCGGCATGTAATGCATGTC*		169	
	*TACAACCCCGATGTGTAAACACA*			
*S. canis*	*AAGGAAATGGAACACGTTAGGG*		924	
	*CTCCTACCATTACCTCTTAAGGTA*			
*S. dysgalactiae*	*AAGGAAATGGAACACGTTAGGG*		1508	
	*TCCTACCATGACACTAATGTGTC*			
*S. parauberis*	*GAA ACT CTT CTA GCA GAC GTG GAA TC*	Superoxide Dismutase A (sod A)	254	[27]
	*CCT TCT TTA TTT ACA ACT AGC CAT GCC*			
*S. uberis*	*TGG CGT TAT TAT CTG ATG TGT CAT CA*		321	
	*CCA AAA TAG GCT GTT TAC CTT CAG AA*			
*S. pyogenes*	*AAAGACCGCCTTAACCACCT*	*S. pyogenes*-specific gene Spy1258	407	[28]
	*TGGCAAGGTAAACTTCTAAAGCA*			

### FTIR spectroscopy

All isolates were streaked onto tryptic soy agar (Oxoid, Basingstoke, United Kingdom) with a drigalski spatula and incubated at 30°C for 24 ± 0.5 hours. A loop-full of cells were suspended in 100 μl distilled water and vortexed. An aliquot of 30 μl was transferred to a ZnSe sample carrier (BrukerOptics GmbH, Ettlingen, Germany) and dried for 40 minutes at 40°C. The FTIR measurements were performed using an HTS-XT microplate adapter coupled to a Tensor 27 FTIR spectrometer (both BrukerOptics GmbH, Ettlingen, Germany). All spectra were recorded between 4000 and 500 cm^-1^ in transmission mode, with a resolution of 6 cm^-1^ and an aperture of 6.0 mm. For each spectrum, 32 scans were averaged. The analysis of data was carried out using the OPUS software (version 6.5; BrukerOptics GmbH, Ettlingen, Germany).

### IR spectral library

A spectral database was established following the approach previously described by Helm et al. [29] using the OPUS software. In brief, IR spectra of unknown microorganisms are identified by comparison with all entries of the spectral library and a distance measure is calculated based on the Pearson’s product moment correlation coefficient. This spectral distance (D-value) is a measure of the dissimilarity between two spectra where zero is a complete match of indistinguishable spectra and 2000 complete dissimilarity. The reference database was built with vector-normalized second derivative spectra.

### Unsupervised hierarchical cluster analysis (UHCA)

For hierarchical cluster analysis spectral windows from 3.000 to 2.800, 1.800 to 1.500, 1.500 to 1.200, 1.200 to 900 and 900 to 700 cm^-1^ with weight factor 1 and repro-level 30 were used. First derivatives were calculated using the Savitzky-Golay algorithm with nine smoothing points and Average linkage algorithm according to the OPUS software (BrukerOptics GmbH, Ettlingen, Germany) was used. UHCA was employed to determine the architecture of the nets for the ANN and to decipher the optimal distribution of the species into the different classes and levels, which is the first step in developing the ANN.

### Artificial neural networks (ANN)

ANNs resemble biological neurons by consisting of an input layer (=dendrites), a hidden layer (=cell nucleus) and an output layer (=axons). The input data are absorption values at specific wavenumbers, the hidden layer processes the data and the output data are predefined classes (e.g. species) that represent final identification results. Since ANNs are supervised classification systems, spectra are split into a training set for calibration, a pre-validation set and a test set for internal validation. The identification procedure is divided into several classification problems that are solved by developing single subnets. The resulting hierarchical ANN consists of all subnets for identifying closely related species like *Streptococcus* spp.

All 210 strains used for the reference dataset were measured ten times independently to include possible biological variances in sampling procedures. For training the ANN, the 2.100 reference spectra were divided into a training set (8 spectra of each strain), a set for internal validation (1 spectrum of each strain) and a test set (1 spectrum of each strain). External validation of the ANN was performed by using 173 strains of gram-positive, catalase-negative cocci unknown to the ANN and not included in the training data set. Strains used for the external validation were measured three times independently. The NeuroDeveloper software (version 2.3; Synthon GmbH, Heidelberg, Germany) was used for calculating ANNs, which gradually lead to an ANN-based classification by means of single combined modules [30]. In a data pre-processing step, the predefined spectral windows between 700 cm^-1^ to 1.800 cm^-1^ and 2.800 to 3.030 cm^-1^ were utilized to select the 100 most discriminative wavelengths using the COVAR algorithm in order to reduce input data to prevent overfitting. Moreover, first derivatives of spectral data were calculated with a 9- point Savitzky-Golay filter. During the training that was carried out by the NeuroSimulator module, all classification levels established were fully connected feed-forward neural networks trained using the Rprop algorithm. The numbers of neurons in the input layer and the hidden layer were continuously increased and the minimum of the validation error was stored. When the internal validation results for each single net obtained > 95% of correct classification, one complete hierarchically structured ANN was compiled by linking all single ANNs. Finally, (unknown) spectra were classified using the ANN established. For classification, following parameters were applied and at least two have to be obtained: WTA (winner takes all), 40-20-40 rule and a potential extrapolation [30]. As identification results three different classifications are possible: (i) correct identification, (ii) incorrect identification, or (iii) failed (unknown classification).

### MALDI – TOF mass spectrometry (MS)

#### *Sample preparation and MALDI-TOF MS*

Half of a loop of bacterial cells grown on tryptic soy agar (TSA, Merck, Darmstadt, Germany) supplemented with Glucose (1 g/l) at 34°C for 48 h was suspended in 300 μl distilled water and vortexed. After adding 900 μl of ethanol (Roth, Karlsruhe, Germany), the suspension was vortexed and cells were harvested by centrifugation for 2 min, 14000 rpm, at RT. The pellets were dried in a concentrator for 15 min at 30° (Concentrator plus, Eppendorf AG, Hamburg, Germany). 50 μl of 70% formic acid (Merck, Darmstadt, Germany) plus 50 μl acetonitrile (Fluka Analytical Sigma-Aldrich, Munich, Germany) were used to dissolve the pellet. After vortexing and centrifugation at 14000 rpm, 2 min, at RT, 1 μl of the supernatant was spotted on a MSP 96 target polished steel plate (Bruker Daltonik GmbH, Bremen, Germany) and air-dried. Subsequently, 1 μl of the matrix-organic solvent solution (HCCA, Sigma-Aldrich, Munich, Germany) was spotted on the spots and allowed to dry at RT. Measurements were carried out with the Microflex LT System, MALDI Biotyper™ (Bruker Daltonik GmbH, Bremen, Germany) equipped with a 60-Hz nitrogen laser, using the Software for FLEX Series 1.3. All spectra were recorded in a linear positive ion detection mode within a mass range from 2.000 to 20.137 Da. The spectrometer settings were as follows: ion source 1 (IS1) 20 kV; ion source 2 (IS2) 16.69 kV; lens voltage: 7 kV; pulsed ion extraction: 150 ns. Each spectrum was created with the software Flex Control software (version 3.0; Bruker Daltonik, GmbH, Germany) and obtained by 240 shots. *Escherichia coli* DH5alpha extract, spiked with two additional proteins (RNase A and myoglobin) provided by Bruker Daltonik GmbH (Bremen, Germany) was used as external calibrator before measurement. A mass accuracy of ± 300 ppm defined a successful calibration. All samples were prepared in duplicates in order to test the reproducibility of the system.

### Spectral data analysis

The analysis of the spectra was carried out using the reference database v3.3.1.0 (Bruker Daltonik, GmbH, Bremen, Germany) containing 4613 entries after an update in October 2012. Log(score) values ranging from 0 to 3 were achieved by matching the unknown isolate with the reference strains (MSP) in the database. A log(score) value ≥ 2.300 indicates a reliable species identification and values between 2.000 to 2.299 represent reliable genus identification. Values between 1.999 and 1.700 refer to probable correct identification on genus level and values below 1.7000 were regarded as non-identifiable.

### Blind study

21 unknown isolates derived from quarter milk samples provided by TgD Styria and Upper Austria were subjected to FTIR spectroscopy and MALDI-TOF MS analysis to validate the practicability and feasibility of both methods. Samples were processed and measured as described above. Species identity of all isolates was confirmed by 16S rDNA sequencing.

## Results

### Strain panels for development of the FTIR identification systems

In total, 363 strains belonging to gram-positive, catalase-negative cocci species, which are commonly known to be associated with bovine mastitis, were isolated from mastitis milk samples. Additional streptococci species were used as out-groups in order to define species borders more precisely and avoid false positive results. In total, the strain set used for the establishment of the spectral library and the ANN comprised 383 isolates (see Table 1).

**Figure 1:**
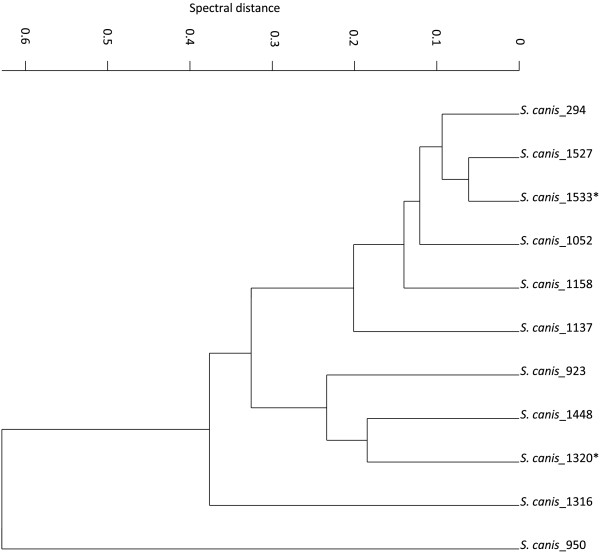
**Hierarchical cluster analysis of *S. canis.*** Hierarchical cluster analysis (HCA) of the first derivatives of FTIR spectra from *S. canis* isolates. Based on the HCA strains were divided into the external validation and training set (for details see text). Spectral region used were 3.000 – 2.800 cm^-1^, 1.800 – 1.500 cm^-1^, 1.500 – 1.200 cm^-1^, 1.200 – 900 cm^-1^ and 900 – 700 cm^-1^. Distances were calculated with normalization to a reproduction level of 30 and the clustering algorithm was Average Linkage algorithm. * indicate strains used for the validation set.

FTIR spectra from all isolates were recorded and processed as described in the Methods Section. Since the coverage of the intraspecies biodiversity is a crucial parameter for building up reference datasets, hierarchical cluster analysis (HCA) of spectral data was performed for each species. HCA provides an overview of spectral similarities among bacterial strains, which makes it a suitable tool for the selection of strains for the reference and the validation set. An example for HCA of *S. canis* spectra is shown in Figure 1. The choice of reference strains needs to span the largest differences in the cluster and validation strains may be selected from different subclusters. Based on the results of HCA, the whole strain panel was divided into a reference (*n* = 210) and validation set (*n* = 173). First, the spectral database was set up using the reference strain set. Thereafter, the validation strain set was used for an independent (external) validation of the established database. All strains used for the external validation were unknown to the IR spectral database and the ANN and not included in the training data set.

### Development of the IR spectral reference database

The reference set (*n* = 210) was used to establish a spectral reference database for the identification of *Streptococcus* spp. and related bacteria. To extract the most specific information out of the entire spectrum (3.000-700 cm^-1^) an iterative process was used. Generally, spectra of microorganisms are divided into five major spectral windows: W1 (3000–2800 cm^-1^) defined as the fatty acid region, W2 (1700–1500 cm^-1^) as the protein region, W3 (1500–1200 cm^-1^) is considered as mixed region of fatty acid and proteins, W4 (1200–900 1200 cm^-1^) contains carbohydrates and W5 (900–700 cm^-1^), is the so called fingerprint region that contains weak but extremely characteristic absorbencies for bacterial isolates [14]. The spectral windows W3 and W4 turned out to be the most suitable ones for the identification and differentiation of *Streptococcus* spp. and related bacteria. For each sample spectrum recorded a list comprising the 12 most similar reference spectra was reported and the identification was considered as valid when the spectral distance of the first hit was ≤ 2 and the first two hits either belonged to the same species or were separated by a spectral distance of at least 0.2.

The final IR database for the identification of gram-positive, catalase-negative cocci compiled by the OPUS software (version 6.5; BrukerOptics GmbH, Germany) included 210 spectra, one for each strain. The combination of spectral windows with highest discriminating power revealed 99.6% correctness of species identification. 1 spectrum was misidentified at species level but 100% correct identification was achieved at genus level. An external validation, using 173 strains, was carried out to test the quality of the database. 96.3% of the strains were correctly identified at species level and 99.0% at genus level (Table 3). All three spectra of one *L. garvieae* isolate and two of three spectra of one *L. lactis* isolate were misidentified as *E. faecalis* and *E. faecium*, respectively. For the species *S. dysgalactiae* and *S. uberis* also some spectra were misidentified.

**Table 3:** **Accuracy of the IR spectral reference database and ANN for the identification of *Streptococcus *** **spp. and related species**

	**ANN**				**Database**				**Combination**			
	**Correct**				**Correct**				**Correct**			
	**Species**	**Genus**	**Failed**	**m.i.**	**Species**	**Genus**	**Failed**	**m.i.**	**Species**	**Genus**	**Failed**	**m.i.**
Known strains (210 spectra)	209 (99.52%)	210 (100%)	0 (0%)	1 (0.5%)	209 (99.6%)	210 (100%)	0 (0%)	1 (0.5%)	210 (100%)	210 (100%)	0 (0%)	0 (0%)
Unknown (519 spectra)	511 (98.5%)	519 (100%)	5 (1%)	3 (0.6%)	500 (96.3%)	513 (99%)	11 (2.2%)	8 (1.4%)	519 (100%)	519 (100%)	0 (0%)	0 (0%)
Total (729 spectra)	720 (98.8%)	729 (100%)	5 (1%)	4 (0.5%)	709 (97.3%)	723 (99.2%)	11 (2.2%)	9 (1.2%)	729 (100%)	729 (100%)	0 (0%)	0 (0%)

m.i.: misidentified.

### Development of the ANN

**Figure 2:**
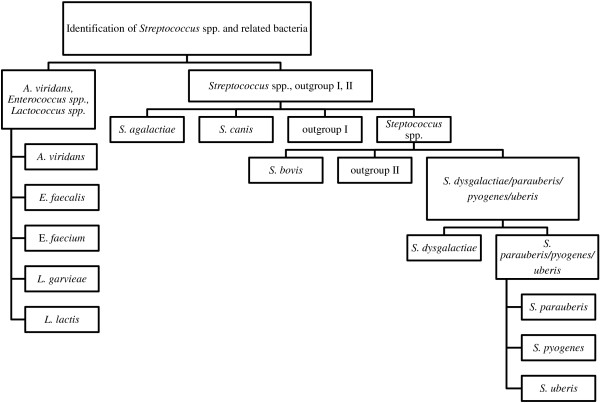
**Artificial neural network (ANN) for the identification of *Streptococcus spp. *and related cocci.** Hierarchical structure for the modular artificial neural network (ANN) for the identification of *Streptococcus spp.* and related cocci. On the first level of the ANN architecture, streptococci are divided from other gram-positive, catalase-negative cocci. On the second level, *A. viridans, Enterococcus spp., Lactococcus spp., S. agalactiae, S. canis,* outgroup I could be identified. The other three levels were used for identifying *S. bovis, S. dysgalactiae, S. parauberis, S. pyogenes, S. uberis* and outgroup II. Species contained in outgroup I and II were used as negative controls and are not associated to bovine mastitis. The following strains were used for outgroup I: *S. mitis, S. pneumoniae, S. sanguinis, S. porcinus, S. gallinarum* and for outgroup II: *Streptococcus equi, Streptococcus gallolyticus* subsp. *macedonicus, Streptococcus suis.*

Since streptococci and related species show a complex population structure, including very closely related species, a multilevel ANN approach was used to establish the FTIR identification system for this group of bacteria. The division of the identification process into several consecutive steps allows the definition of optimal parameters for each identification step, which often leads to an improvement of identification accuracy. For this purpose, HCA from all reference strains was carried out in order to group IR spectra on the basis of their overall similarity. Based on the clusters, all species were divided into groups or single classes for training the ANNs. Groups of species were separated into single classes in a following step. For each ANN an individual choice of input data (wave numbers) optimized to the problem to solve was used and the final ANN comprised a total of 6 connected single subnets distributed over five consecutive levels (Figure 2). At the first level, strains were grouped into two main classes, separating streptococci from the other genera. While *A. viridans*, *Enterococcus* spp. and *Lactococcus* spp. could be separated at the second level as well as *S. agalactiae*, *S. canis* and outgroup I (comprising *Streptococcus mitis, Streptococcus pneumoniae, Streptococcus sanguinis, Streptococcus gallinarum*), the remaining streptococci (including outgroup II) required three additional levels to be split into single species. The overall structure of the ANN is shown in Figure 2.

### Validation of the ANN

The accuracy of the established ANN was validated by an internal validation test as well as an external validation test. The generalization capability of the ANN (internal validation) was examined using one spectrum of each strain not included in the training process. In principle, three classification results are possible: correct classification, misclassification, and failed class assignment. The internal validation using 210 spectra resulted in 99.5% correct classifications (Table 3). Only one single spectrum out of the total of 210 spectra was misclassified. The external validation, including only strains not used for the network development, underpinned the robustness and suitability of this classification system. A total of 519 spectra from 173 strains (three spectra for each strain) were recorded and a correct identification was observed for 98.5% of the spectra. Only three spectra (0.6%) were misidentified and 1% failed class assignment. In the latter case the ANN could not decide to which class the spectrum belong (Table 3). These results demonstrate the strong potential and reliability of the network considering that only a total of three spectra from two different species (*S. parauberis*, *S. uberis*) were misclassified.

A further improvement of the FTIR identification system was achieved by combining the reference database and the ANN. All 19 spectra misidentified by the IR database were correctly identified by the ANN. For application in routine analyses, results of the ANN should be given priority, as the overall accuracy is higher. For samples that failed classification by the ANN, the spectra should be reanalyzed using the spectral database since all spectra that failed identification by ANN were identified correctly by the FTIR spectral database. The accuracy values clearly demonstrated that the microbiodiversity of the gram-positive, catalase-negative cocci were covered by the FTIR library and represented by the artificial neural network.

### MALDI-TOF identification of gram-positive, catalase-negative cocci

MALDI-TOF MS in combination with a commercially available MS spectral database (MALDI, Biotyper software version 3.1) was employed to analyze a subset of mastitis associated streptococci and related cocci used in this study. 210 representative isolates were selected from the strain panel, which comprises a total of 383 strains. 200 out of the 210 isolates (95.2%) were identified correctly at species level (log(score) value ≥ 2.3), 100% at the genus level (Table 4). *S. canis, S. dysgalactiae* and *S. pyogenes* formed a distinct cluster within the *Streptococcus* group and particularly *S. canis* and *S. dysgalactiae* were not always unequivocally identifiable. Two strains of *S. dysgalactiae* could not be clearly differentiated from *S. canis* and one strain of *S. canis* could not be distinguished from *S. dysgalactiae.* The type strain of *S. bovis* was misidentified as *Streptococcus lutetiensis* and one strain of *S. dysgalactiae* was misidentified as *S. canis.*

**Table 4:** **Accuracy of MALDI-TOF mass spectrometry for the identification of *Streptococcus *** **spp. and related species**

**Species**	**No of strains**	**Correct species identification log(score) value ≥ 2.3**	**Probable species identification log(score) value 2.0-2.299 0.15 distance between single species**	**Correct genus identification log(score) value 2.0-2.299 < 0.15 distance between single species**	**Mis-identification**^**#**^
*A. viridans*	6	4	2 (33.3%)		
*E. faecalis*	35	35			
*E. faecium*	6	6			
*L. garvieae*	6	5	1 (16.6%)		
*L. lactis*	11	11			
*S. agalactiae*	11	11			
S. gallolyticus/bovis*	6	5			1 (16.6%)
*S. canis*	6	5		1 (16.6%)	
*S. dysgalactiae*	36	33		2 (5.5%)	1 (2.8%)
*S. parauberis*	6	4	2 (33.3%)		
*S. pyogenes*	11	11			
*S. uberis*	70	70			
Total	210	200	5 (2.4%)	3 (1.4%)	2 (1%)

^#^Misidentification only at species level, all isolates were correctly identified by genus level. *S. bovis is indicated as *S. gallolyticus* in the MALDI-TOF reference database. One strain was misidentified as *Streptococcus lutetiensis.*

A subset of 231 strains derived from the strain panel, generated for this study (see Table 1), was used for this validation approach. Results were analyzed following the score value system according to Bruker Daltonik GmbH (Bremen, Germany). Log(score) values from 3.00 to 2.30 indicate reliable species identification. Log(score) values from 2.29 to 2.00 indicate reliable genus and probable species identification, if a score value distance of at least 0.15 between the two best-scored species is given (Huber et al., unpublished).

### Blind study

In order to obtain accurate identification systems for routine diagnosis, 21 unknown isolates originating from mastitis milk samples were used (i) to test the efficiency and robustness of spectroscopic and spectrometric methods applied, and (ii) to test if all mastitis relevant *Streptococcus* spp. and related species were covered by the ANN and reference database. All strains were measured and analyzed by FTIR and MALDI-TOF without pre-identification by conventional methods. The FTIR based ANN classification yielded 100% correct species identification as well as the IR reference database. A success rate of 90.5% (log(score) value ≥ 2.3) was achieved for species identification by MALDI-TOF MS. Correct species identification was confirmed by 16S rRNA gene sequencing (Table 5).

**Table 5:** FTIR and MALDI-TOF MS identification results of blind study

**No of isolates**	**16S rRNA sequencing**	**Identification accuracy**		**Comment**
		**FTIR ( *n *)**	**MALDI-TOF MS( *n *) Log(score) value ≥2.3**	
1	*A. viridans*	1/1	1/0	Match (0%)
3	*E. faecalis*	3/3	3/3	Match (100%)
2	*L. garvieae*	2/2	2/1	Match (50%)
1	*L. lactis*	1/1	1/1	Match (100%)
3	*S. dysgalactiae*	3/3	3/3	Match (100%)
11	*S. uberis*	11/11	11/11	Match (100%)
21		21/21	21/19	Match (90.5%)

Blind validation study to assess the suitability of FTIR and MALDI-TOF MS for routine diagnosis of mastitis associated *Streptococcus* spp. and related species. 21 isolates, provided by TgD Styria and Upper Austria, were used for this blind test.

## Discussion

A rapid, inexpensive and easy handling method for identification of mastitis pathogens is an essential task in veterinary routine diagnosis and a prerequisite for targeted (antibiotic) treatment and efficient prophylaxes.

Especially, the identification of gram-positive, catalase-negative cocci at species level, in particular, still represents a major challenge. Wyder et al. [2] and McDonald et al. [4] clearly showed that an unequivocal identification of *Streptococcus* spp*.* and related mastitis associated cocci, especially minor pathogens such as *L. garvieae*, is difficult by methods currently used in routine diagnosis. We therefore assessed the potential of FTIR spectroscopy and MALDI-TOF MS for identification and differentiation of mastitis relevant gram-positive, catalase-negative cocci. FTIR spectroscopy was already successfully applied for the classification of human pathogenic enterococci and food related lactic acid bacteria ([31,32]) and MALDI TOF MS is becoming more and more prominent in human clinical diagnosis [20,22]. The strain panel generated for this study comprised a total of 383 isolates, belonging to 21 species and four genera. In addition to the most important *Streptococcus* species, other mastitis-associated gram-positive, catalase-negative cocci that occur less frequently but have an intramammary origin, such as *L. garvieae* or *S. parauberis*, were included (Table 1).

Since many of these species are phylogenetically closely related, their differentiation and identification is often challenging. For this reason, a spectral reference database and FTIR based ANNs were established and combined to ensure fast and accurate bacterial identification at species level. The reference database, which averages all spectral differences into one distance measure (D-value), is generally a very robust system but it does not provide as much flexibility as ANN [10]. Since the selection of appropriate spectral regions for identification of microbes is known to be species specific [12,15], care was taken to select spectral regions providing the highest discriminatory power for classification and identification of *Streptococci* and related species. The spectral regions between 1.500 to 1.200 and 1.200 to 900 cm^-1^, covering the mixed region and the polysaccharide region, were found to be best suited for the identification and discrimination of gram-positive, catalase-negative cocci.

The ANN detects small differences in biodiversity patterns due to the selection of single wavenumbers in contrast to whole spectral regions as chosen for the library, which explains its higher discriminatory power and robustness compared to the spectral library based identification system. The selection of specific wavenumbers by the ANN ensures that only the most significant information is extracted and put into the training process. The complete net consisted of single nets that are highly discriminatory for the defined classes on the basis of the most discriminative wavenumbers [30]. During the training process, only significant information is extracted, trained and the differences between the spectra used for calibration are stored for achieving high identification accuracy [14]. The final net established in this study was highly discriminatory and provided highly specific and reliable results. For instance, during the external validation, using strains unknown to the ANN, an overall identification correctness of 98.5% at species level was achieved. Although most of the species could be easily separated in three steps (levels) the discrimination of *S. dysgalactiae*, *S. parauberis, S. pyogenes* and *S. uberis* required two additional levels. This might be due to similar spectral patterns that are used for species identification, particularly noticeable for *S. uberis* and *S. dysgalactiae*. By combining the ANN with the reference database 100% correct identification was achieved, underpinning the specificity and robustness of FTIR-based identification system developed in this study. It is assumed that FTIR spectroscopy combined with chemometrics could significantly improve the diagnosis of mastitis-associated streptococci and related cocci. Indeed, results from a blind study, including isolates from quarter milk samples sent to routine mastitis diagnostic labs in Austria, confirmed the principal suitability of the established FTIR-systems for routine diagnosis (Table 5).

Another particular advantage of FTIR is its high discriminatory power, allowing discrimination of bacterial subtypes and its use for epidemiological studies and determination of contamination sources [9,13,17]. The latter applications could be very important concerning therapy control, prophylaxes and detection of source of infection [33].

As a second physico-chemical method we included MALDI-TOF MS in this study since it is already established in clinical microbiology but is still rarely used for routine diagnosis in veterinary medicine. For instance, Barreiro et al. [18] showed that MALDI-TOF is, in principle, suitable for identification of subclinical mastitis pathogens. We therefore used a subset of strains (n = 210) derived from the strain panel, generated for the development of the FTIR identification system, to test the performance of MALDI-TOF MS. Unequivocal results (log(score) values > 2.3) were achieved for 95.2% of the strains and about 2.4% of the strains showed log(score) values between 2.0 and 2.3. Distinguishing *S. dysgalactiae* from *S. canis* and *S. bovis* from *S. bovis/equinus* complex turned out to be difficult by MALDI-TOF MS but not by FTIR spectroscopy*.* For instance, one *S. dysgalactiae* and one *S. bovis* were even misidentified by MALDI-TOF MS (Table 4)*.* These results might be explained by the close phylogenetic relationship of the aforementioned species to *S. pyogenes* and *S. equinus*, respectively [34,35]. Moreover it must be taken into consideration that the taxonomy of the *S. bovis/equinus* complex is very complex and has undergone several changes over time, resulting in the reassignment of *S. bovis* biotype I to *S. gallolyticus* (for overview see [34]). Furthermore, the MALDI BioTyper database (V.3) only includes reference MS spectra from six *S. lutentiensis,* two *Streptococus equinus* and one *Streptococcus infantarius* strains but no reference MS spectra from *S. bovis* strains. Limited resolution of MALDI-TOF MS for closely related *Streptococcus* spp. was also reported from a recent study of Raemy et al. [36]. Some isolates, especially those belonging to *S. dysgalactiae*, could only be identified at genus level and isolates belonging to the *S. mitis/oralis/pseudopneumoniae* group could not be further discriminated by using the SARAMIS software (see [36], Figure 2). Identification and discrimination of bacterial species by MALDI-TOF MS is mainly based on ribosomal proteins whereas FTIR spectroscopy covered the entire biochemical composition of a cell explaining the higher discriminatory power of the latter technique. In the study presented, the carbohydrate region contributed significantly to the successful identification of streptococci by FTIR. Nevertheless, both methods tested are suitable for the identification of gram-positive, catalase-negative cocci. In contrast to FTIR, MALDI-TOF MS does not require specific culture conditions, which makes it easier to implement it in routine diagnosis, although at the cost of resolution power. The results of the blind study strengthen the assumption that the methods presented in this work are interesting novel tools for routine diagnosis. Notably, *A. viridans*, *L. garvieae* and *L. lactis* represented about 20% of the blind study isolates, suggesting that these so called “minor pathogens” occur more frequently than previously thought. These results are in line with recent findings of Wyder et al. [2], who also reported similar frequencies of *A. viridans* and *L. garvieae* in quarter milk samples from Swiss dairy cows. In order to avoid misclassification and to get validated data on the prevalence of the ‘major mastitis causing’ *Streptococcus* spp. as well as on other gram-positive, catalase-negative cocci associated with bovine mastitis, reliable and robust identification techniques are of utmost importance and urgent need.

## Conclusions

In summary our results show that both FTIR spectroscopy and MALDI-TOF MS are suitable for the identification and differentiation of mastitis associated *Streptococcus* species and related genera. Both methods are straight forward, allowing a robust and accurate identification of bacteria down to species level. Due to its higher discriminatory capacities, FTIR has also the potential to be used for biotyping of mastitis pathogens at a subspecies or strain level and may also represent an interesting tool for the determination of contamination sources and infection routes in dairy farming. Compared to conventional microbiological methods, both techniques provide fast, accurate identification results by low operating costs, although FTIR spectroscopy was slightly superior to MALDI-TOF-MS. A rapid and unequivocal bacterial identification could not only facilitate earlier and targeted treatment of mastitis but could also improve biosafety monitoring of raw milk.

## Competing interests

The authors declare that they have no competing interests.

## Authors’ contributions

LS contributed to the design of the study, conducted all experiments and drafted the manuscript. MW participated in the FTIR study design, developed together with LS the concept for the artificial neural network and contributed to the manuscript finalization. IH was involved in the design and data analysis of the MALDI-TOF experiments. MES conceived and coordinated the study, and contributed to completion of the manuscript. All authors read and approved the final manuscript.

## Supplementary Material

Additional file 1 Reference strains used in Schabauer et al. ‘Novel physico-chemical diagnostic tools for bovine mastitis associated gram-positive, catalase-negative cocci’. Click here for file
