# Supplementary material for: Novel physico-chemical diagnostic tools for high throughput identification of bovine mastitis associated gram-positive, catalase-negative cocci
Source: BMC Vet Res. 2014 Jul 11;10:156. doi: 10.1186/1746-6148-10-156 (PMC4105049; doi:10.1186/1746-6148-10-156)
Supplement: Additional file 1 — Reference strains used in Schabauer et al. ‘Novel physico-chemical diagnostic tools for bovine mastitis associated gram-positive, catalase-negative cocci’. [file 1746-6148-10-156-S1.pdf]

Additional file 1 – Reference strains used in this study

Strain	Species	DSMZ/ATCC
**Mastitis associated reference strains (*n* = 16)**		
1478	*Aerococcus viridans*	20340 T
1463	*Enterococcus faecalis*	ATCC 51299
1476	*Enterococcus faecalis*	20478 T
1462	*Enterococcus faecium*	ATCC 700221
1464	*Enterococcus faecium*	20477 T
1081	*Lactococcus garvieae*	20064
1084	*Lactococcus garvieae*	20684 T
1137	*Lactococcus lactis*	20481 T
1426	*Streptococcus agalactiae*	2134 T
1138	*Streptococcus bovis*	20480 T
1136	*Streptococcus canis*	20715 T
1135	*Streptococcus dysgalactiae*	20662 T
1127	*Streptococcus pyogenes*	2071
1429	*Streptococcus pyogenes*	20565 T
1477	*Streptococcus parauberis*	6631 T
1214	*Streptococcus uberis*	20569 T
**Reference strains used for the outgroup (*n* = 6)**		
1428	*Streptococcus equi subsp. equi*	20561 T
1431	*Streptococcus equi subsp. zooepidemicus*	20727 T
1090	*Streptococcus mutans*	20523 T
1427	*Streptococcus pneumoniae*	20566 T
1109	*Streptococcus porcinus*	20725 T
1430	*Streptococcus suis*	9682 T
